# Towards screening Barrett’s oesophagus: current guidelines, imaging modalities and future developments

**DOI:** 10.1007/s12328-020-01135-2

**Published:** 2020-06-03

**Authors:** Ishaan Maitra, Ravindra Sudhachandra Date, Francis Luke Martin

**Affiliations:** 1 School of Pharmacy and Biomedical Sciences, University of Central Lancashire, Preston, PR1 2HE UK; 2 Lancashire Teaching Hospitals NHS Foundation Trust, Royal Preston Hospital, Preston, PR2 9HT UK; 3 Biocel Ltd, Hull, HU10 7TS UK

**Keywords:** Barrett’s oesophagus, Imaging modalities, Oesophageal adenocarcinoma, Screening

## Abstract

Barrett’s oesophagus is the only known precursor to oesophageal adenocarcinoma (OAC). Although guidelines on the screening and surveillance exist in Barrett’s oesophagus, the current strategies are inadequate. Oesophagogastroduodenoscopy (OGD) is the gold standard method in screening for Barrett’s oesophagus. This invasive method is expensive with associated risks negating its use as a current screening tool for Barrett’s oesophagus. This review explores current definitions, epidemiology, biomarkers, surveillance, and screening in Barrett’s oesophagus. Imaging modalities applicable to this condition are discussed, in addition to future developments. There is an urgent need for an alternative non-invasive method of screening and/or surveillance which could be highly beneficial towards reducing waiting times, alleviating patient fears and reducing future costs in current healthcare services. Vibrational spectroscopy has been shown to be promising in categorising Barrett’s oesophagus through to high-grade dysplasia (HGD) and OAC. These techniques need further validation through multicentre trials.

## Introduction

Oesophageal adenocarcinoma (OAC) has now replaced squamous cell carcinoma (SCC) as the most common type of oesophageal malignancy in the Western world [1, 2]. OAC is aggressive and usually presents late with a poor prognosis with an overall 5-year survival below 25% [3]. Despite technological enhancements related to preventative strategies and more effective combination therapies, the overall incidence of oesophageal adenocarcinoma has risen [4].

There is a proven association between adenocarcinoma and Barrett’s oesophagus, a condition that appears to arise in response to chronic inflammation from gastro-oesophageal reflux disease (GORD) [5]. Reflux induces metaplasia, which in turn leads to high-grade dysplasia (HGD) and invasive OAC. Barrett’s oesophagus is the only known precursor to OAC to date and has a small prevalence in the European population (up to 2%) [6]. General surveillance through oesophagogastroduodenoscopy (OGD) of all individuals with Barrett’s oesophagus is not cost-effective as the annual incidence of oesophageal adenocarcinoma developing in Barrett’s is only 0.33% [7, 8]. This highlights the need to adapt surveillance programs to include individuals with Barrett’s at high absolute risk of tumour progression.

There remains a lack of consensus regarding the natural history of Barrett’s oesophagus [9]. Furthermore, there is a lack of reliable predictive biomarkers that might enable us to risk stratify Barrett’s oesophagus patients [10, 11]. Prospective studies have not established a survival benefit for screening and surveillance [9]. All present guidelines regarding screening, surveillance and management fail to demonstrate clear evidence for an established benefit and cost-effectiveness of surveillance, and robust risk stratification for patients to best use health resources [12].

### Historical perspective and definition

Australian-born surgeon Norman Rupert Barrett first developed the term ‘Barrett’s Oesophagus’ in patients with ulcerations in a tubular organ suggestive of an oesophagus, but whose distal, ulcerated portion was lined by columnar epithelium [13]. Bosher and Taylor elaborated on Mr. Barrett’s definition describing intestinal type goblet cells in the columnar-lined oesophagus in 1951 [14]. Morson and Belcher reported the first case of malignancy related to Barrett’s oesophagus in 1952. The case was of a patient who developed an adenocarcinoma in oesophageal mucosa that presented ‘atrophic changes with a tendency towards intestinal type containing many goblet cells [15].

The definition of Barrett’s oesophagus is controversial and worldwide professional societies have different definitions related to endoscopic documentation of columnar-lined mucosa (Table 1). The presence of specialised intestinal metaplasia (SIM) characterised by the presence of goblet cells is important in its definition. SIM is associated with the risk of progression to low-grade dysplasia (LGD), high-grade dysplasia (HGD) and adenocarcinoma (OAC).

**Table 1:** Worldwide professional societies’ definitions of Barrett’s oesophagus

	ACG (USA) [9]	BSG (England) [16]	AGA (USA) [17]	SFED (France) [18]
Intestinal metaplasia (biopsies)	Yes	Yes	Yes	Yes
Endoscopic documentation of columnar-lined mucosa	Yes	No	Yes	Yes

*ACG* American College of Gastroenterology, *BSG* British Society of Gastroenterology, *AGA* American Gastroenterological Association, *SFED* Société Française d'Endoscopie Digestive

### Endoscopic evaluation

The significant anatomical landmark in Barrett’s oesophagus identification is the oesophagogastric junction (OGJ). This is usually identified as the proximal extent of the upper gastric folds. The squamocolumnar junction (Z line) is the point at which squamous mucosa of the oesophagus meets the columnar mucosa of the stomach. Irregularity of the Z line is defined as <1 cm non-circumferential tongues of columnar mucosa at the squamocolumnar junction. This is not currently defined as Barrett’s oesophagus [16] but in up to 40% of such cases histopathology will reveal intestinal metaplasia (IM).

**Fig. 1:**
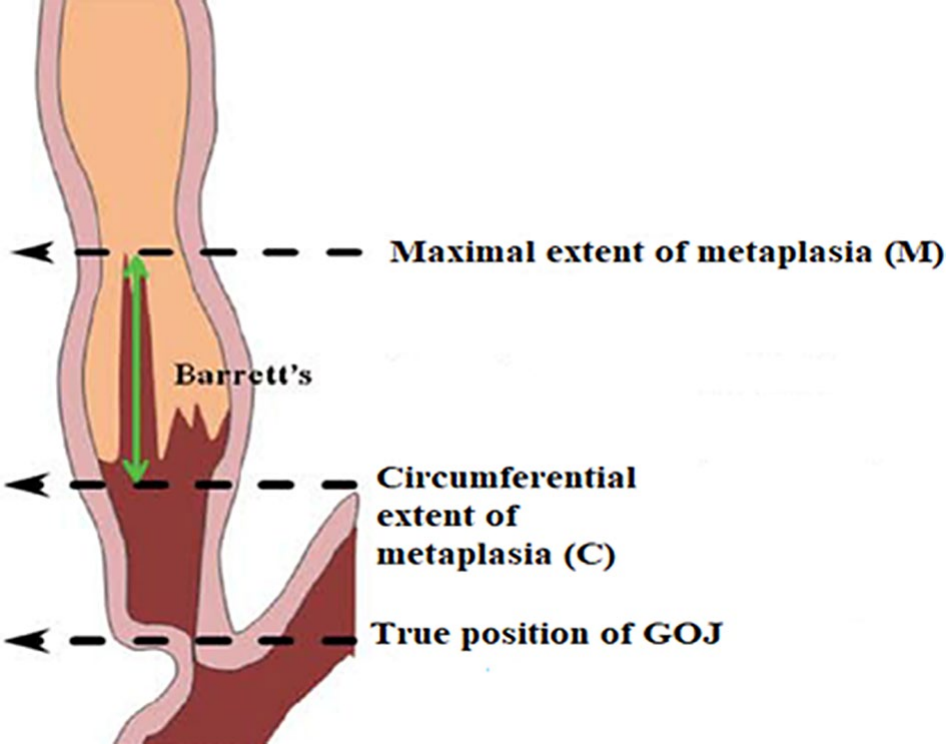
Illustration showing ‘C’ and ‘M’ measurements. Adapted from https://www.endoscopy-campus.com/en/klassifikationen/prag-klassifikationen-des-barrett-oesophagus/ (accessed 16th October 2019)

Barrett’s oesophagus was initially characterised into short- and long-segment disease (< or >3 cm) based on endoscopic metaplastic epithelial findings [19]. The Prague ‘C’ and ‘M’ classification takes the circumferential (C) and maximum (M) tongue extent of the endoscopically visualised Barrett’s above the OGJ into account (Fig. 1; Table 2).

**Table 2:** Vienna classification of epithelial neoplasia of the digestive tract [45]

Category	Classification
1	Negative for neoplasia/dysplasia
2	Indefinite for neoplasia/dysplasia
3	Non-invasive low-grade neoplasia
	Low-grade adenoma/dysplasia
4	Non-invasive high-grade neoplasia
4.1	High-grade adenoma/dysplasia
4.1	Non-invasive carcinoma (carcinoma in situ)
4.1	Suspicion of invasive carcinoma
5	Invasive neoplasia
5.1	Intramucosal carcinoma
5.2	Submucosal carcinoma or beyond

**Fig. 2:**
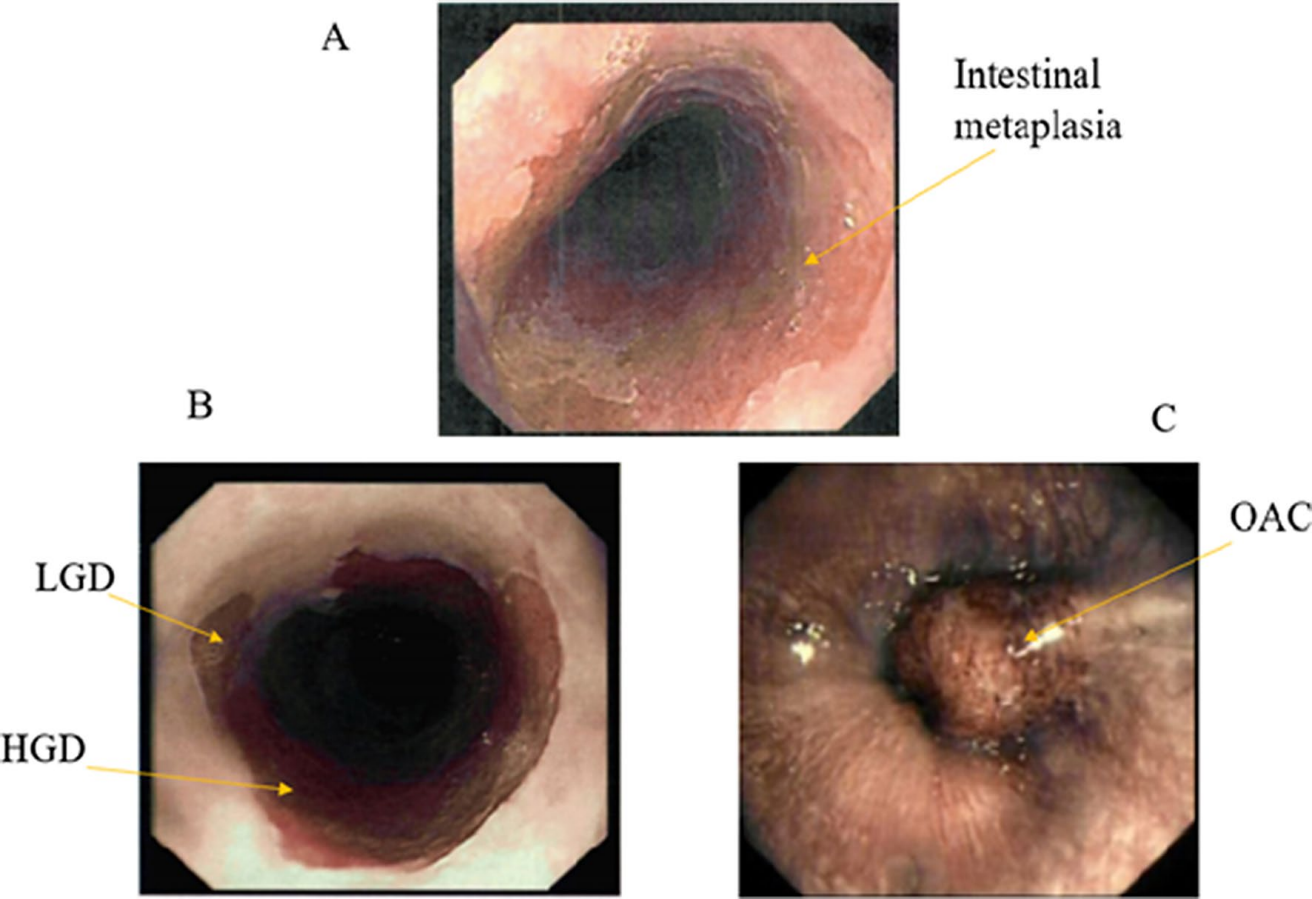
Endoscopic visualisation of **a** Barrett’s segment, **b** Barrett’s with LGD and HGD, **c** OAC

The sensitivity of endoscopic examination for the diagnosis of Barrett’s oesophagus increases with increasing length [20]. The overall prevalence of short-segment Barrett’s oesophagus is greater than long-segment Barrett’s oesophagus in studies where biopsies are systematically taken using established protocols [21]. The overall reliability of endoscopic examination and biopsy is approximately 80%. Given the inter-observer variability of diagnosing short segments, especially those <1 cm, and the exclusion of this length from some studies, it is uncertain whether segments <1 cm are associated with an increased risk of OAC (Fig. 2) [22].

### Risk factors and epidemiology

Males in their 6th decade of life are twice as likely to have a diagnosis of Barrett’s oesophagus than females [23]. White subjects have a 4–6 times higher incidence of the disease compared to black subjects [24]. Other risk factors such as chronic GORD, reduced *Helicobacter pylori* and central obesity also increase the risk of Barrett’s oesophagus in multiple studies [25]. Alcohol consumption does not appear to be a strong risk factor.

The worldwide increase in GORD is accompanied by a rapid increase in prevalence of Barrett’s oesophagus, the main target for prevention of OAC [25]. The exact prevalence in different populations is difficult to assess as the condition is asymptomatic, and a diagnosis is made only when an endoscopy is performed [26]. The latter is usually performed for persistent GORD symptoms [26]. The prevalence of Barrett’s oesophagus in the unselected general population is between 1 and 2% in European studies and approximately 5–6% in the United States [27].

Further population-based studies have provided further insight into the prevalence of Barrett’s oesophagus. The Swedish *Kalixanda* study found that up to 10.3% of patients with GORD symptoms had an endoscopic Barrett’s oesophagus segment [6]. In an Italian study of 1033 patients, histology confirmed the presence of columnar epithelium in 3.6%, while specialised intestinal metaplasia was observed in 1.6% of them [3]. Further UK-detailed studies on age- and sex-related distribution of Barrett’s oesophagus prevalence have observed that the prevalence of Barrett’s oesophagus increased with 7.4% for each additional year of age between the age of 20 and 59 year in males. It showed a similar pattern though with a 20-year delay in the female population [28]. In a meta-analysis of 23 studies from Asia, the prevalence of endoscopically diagnosed Barrett’s oesophagus was 7.8% with histologically confirmed Barrett’s in 1.6% [29]. The prevalence of LGD, HGD and adenocarcinoma in cases with histologically proven BO was 6.9%, 3.0 and 2.0%, respectively [29]. In studies from the United States of America rates are 1.3–9.8%, 0–5.3% and 1.3–5.7%, respectively [30].

### Natural history and progression to OAC

It has been shown that specialised intestinal metaplasia arising in Barrett’s oesophagus is a risk factor for progression to cancer. A meta-analysis from 2012 including 57 studies (*n* = 11,434 patients) reports an annual incidence of oesophageal adenocarcinoma developing in Barrett’s oesophagus of 0.33% (95% CI 0.28–0.38%) [7]. Patients with Barrett’s oesophagus are ten times more likely to die from other causes than oesophageal cancer. It appears that men progress to cancer at twice the rate of women, patients with short-segment Barrett’s oesophagus are least likely to progress and those with dysplasia at index biopsy are the most likely to progress [7].

Progression rates to dysplasia and adenocarcinoma were initially established from a Dutch cohort study in Rotterdam. One hundred and sixty-six patients were recruited from 1973 to 1983, and endoscopic surveillance was started from 2001. Thirteen patients (M/F 10/3) developed HGD or OAC during follow-up. These were all symptomatic cases of HGD/OAC as the patients were not under endoscopic surveillance and were only reinvestigated for symptoms. These cases were observed over a period of 1967 patient years, 1 per 151 years of follow-up or 0.66 per annum (95% CI 0.58–0.74) [31].

Subsequent meta-analyses and systematic reviews from 2007 to 2017 report progression rates to adenocarcinoma ranging from 2.2 to 6.3 per 1000 patient years when focusing on all patients with Barrett’s oesophagus [32–34] Singh et al*.* [30] conducted a systematic review of 24 studies (*N* = 2694). This concluded an annual progression rate of Barrett’s oesophagus to adenocarcinoma of 5.4 (3.2–7.6) per 1000 years.

Current literature is reliant upon a single pathologist’s interpretation of the histology. Since the diagnosis of dysplasia is investigator dependent, more rigorous criteria ask for confirmation of dysplasia by a second histopathologist. Subsequently, a proportion of patients are down-staged which may affect epidemiological studies [26]. Kestens et al*.* [35] re-assessed low-grade dysplastic samples of 231 Barrett’s oesophagus patients. LGD was confirmed in 161 (70%); the remainder was mostly downgraded to no dysplasia or indefinite for dysplasia. A recent systematic review and meta-analysis conducted in 2017 confirmed that the risk of progression to HGD or OAC in Barrett’s oesophagus patients were primarily determined by the presence or absence of LGD [OR 4.2 (2.1–8.5)] [36].

Patients with GORD can subsequently develop Barrett’s oesophagus and go on to develop LGD, HGD and OAC. At an early stage, these conditions can be treated by ablative and minimally invasive techniques with limited risk. However, at an advanced stage, OAC requires invasive treatment with considerable burden, financial cost, and mortality [26]. Early detection and prevention are the key strategies to manage OAC. The argument as to which Barrett’s oesophagus patients are most likely to benefit from surveillance and management centres on the high prevalence of Barrett’s oesophagus and the low cancer incidence amongst Barrett’s oesophagus cases. This should be weighed up against the burden of invasive treatment and the high mortality in OAC [26].

With the overall risk of OAC in Barrett’s oesophagus being low, patients are often middle-to-older ages with obesity and metabolic syndrome. Many patients will succumb to another condition. This is often not conveyed to patients and many live-in fear of developing malignancy disproportionate to their definite risk. This point further highlights the need to adapt surveillance programs to include individuals with Barrett’s at high absolute risk of tumour progression.

### Histopathology

For a non-suspicious segment undergoing routine surveillance, mapping biopsies should be taken at 2-cm intervals from each quadrant as well as separate biopsies from the anatomic cardia. For dysplastic segments, biopsies should be taken at 1-cm intervals. This so-called Seattle protocol increases the yield of both low-grade and high-grade dysplasia by 17 and 3%, respectively, compared with random biopsies [37]. A minimum of eight biopsies are required to provide an adequate degree of histological confirmation in Barrett’s oesophagus [38].

In patients with Barrett’s oesophagus <3 cm and no metaplasia or dysplasia, a repeat endoscopic assessment with quadrantic biopsies is recommended as an extra measure to try to establish the diagnosis, as only patients with SIM should undergo further surveillance [16]. In patients with long-segment disease (>3 cm), SIM is almost always found. Targeted biopsies from any lesions and the immediately surrounding epithelium should be taken separately. Targeted biopsies alone are not a substitute for mapping biopsies as up to 20% of lesions may not be easily visible with white light endoscopy. Histopathological definitions of Barrett’s oesophagus vary worldwide [39]. The requirement of SIM has been supported by studies that have claimed intestinal metaplasia to be a prerequisite for the development of adenocarcinoma. Goblet cells may be identified on routine histological stains (haematoxylin and eosin, H&E), although many institutions routinely employ PAS-Alcian Blue stains to highlight acidic mucin in goblet cells.

Intestinal metaplasia of gastric cardia does not compare to Barrett’s oesophagus and may be less likely to progress to dysplasia [40, 41]. A diagnosis of Barrett’s oesophagus, therefore, cannot be made histologically when the exact site of biopsy of the metaplastic fragment is not known. Barrett’s oesophagus is defined as a clinicopathologic diagnosis with the biopsy taken from an endoscopically visualised salmon-coloured irregularity in the oesophagus. SIM may be either “complete” (when goblet cells are accompanied by absorptive and/or Paneth cells) or “incomplete” (absence of absorptive and/or Paneth cells) [11].

**Fig. 3:**
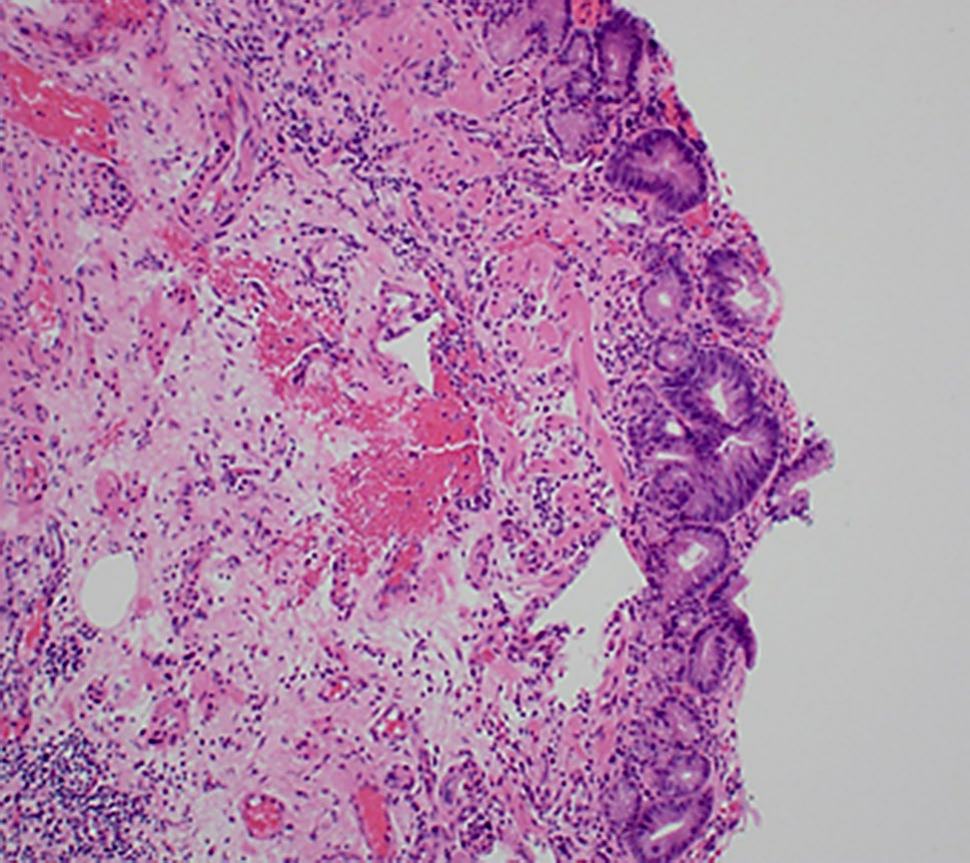
Photomicrograph of intestinal metaplasia (H&E ×100 objective)

Intestinal metaplasia shows inflammatory changes and becomes dysplastic. Dysplasia is assessed in columnar mucosa, and biopsies are categorised as being ‘negative for dysplasia’ if the cells show maturation towards the surface in the form of decreasing nuclear size, decreasing nuclear hyperchromasia and increasing cytoplasmic volume [11]. There may be histological changes deemed insufficient to characterise as dysplasia, categorised as ‘indefinite for dysplasia’ (Fig. 3).

**Fig. 4:**
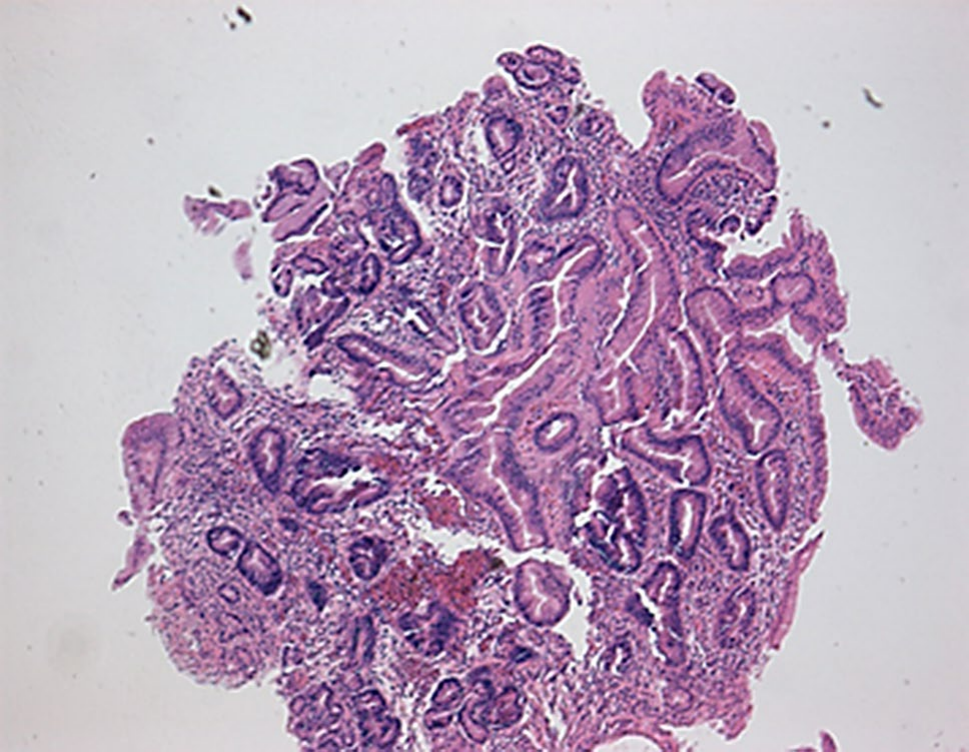
Photomicrograph of low-grade dysplasia (H&E ×100 objective)

Barrett’s adenocarcinoma develops through a multistep process with progressive worsening of a precursor lesion defined as dysplasia. In LGD, the molecular architecture is preserved or minimally abnormal, nuclei are elongated and crowded at the base but not at apex of cells, pseudo-stratification may be extensive, there may be nuclear enlargement, and surface villous transformation may be present [11]. The differentiation of LGD from non-dysplastic tissue is difficult to establish with regards to subjective histopathological criteria (Fig. 4).

**Fig. 5:**
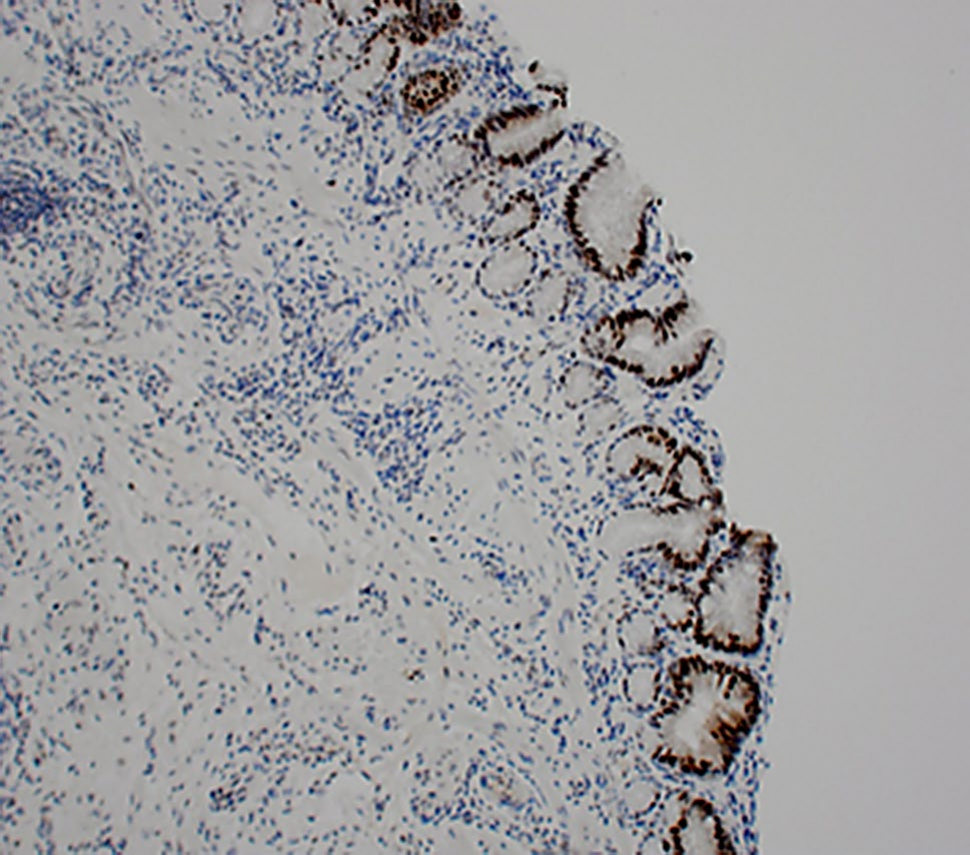
Photomicrograph of high-grade dysplasia (H&E ×100 objective)

**Fig. 6:**
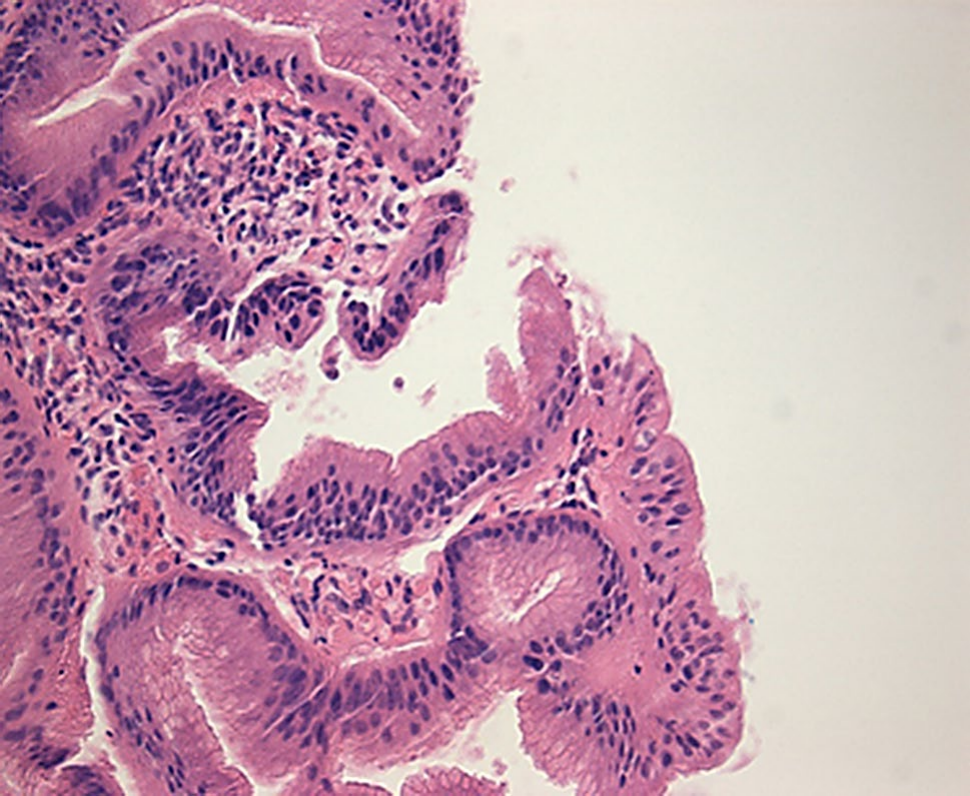
Photomicrograph of oesophageal adenocarcinoma (H&E ×100 objective)

High-grade intestinal type dysplasia demonstrates markedly atypical features including cytologic atypia, nuclear stratification to surface of cell with loss of polarity, and nuclei which are no longer radially oriented [42]. OAC shows marked atypia with no radially orientated nuclei (Figs. 5, 6).

Progressive histological changes are subtle hence resulting in intra- and inter-observer variation in the diagnosis of dysplasia in Barrett’s oesophagus. There are no clearly defined cut-off points that distinguish disease progression when comparing LGD and HGD. Furthermore, sampling errors can occur with small dysplasia sizes and its patchy distribution. Literature from this century has compared general and specialist gastrointestinal pathologists. In a Dutch study by Kerkhof et al. [43], general histopathologists were found to over diagnose HGD. Up to 40% of patients initially diagnosed with HGD by a general pathologist were downgraded (11% no dysplasia, 12% indefinite for dysplasia, 16% LGD) when the samples were reviewed by three experienced gastrointestinal (GI) pathologists [43].

Montgomery et al. [44] rigorously tested criteria, assessing intra-observer and inter-observer reproducibility. When a four-tier grading system was employed (non-dysplastic/indefinite and low grade/high grade/cancer), the kappa index was low (0.43). Kappa improved (0.66) when a simplified classification was used (non-dysplastic/indefinite and low grade/high grade and cancer). These results emphasise the need to obtain a second opinion on problematic cases [44]. Geographical discrepancies are also highlighted in the literature. In a study comparing Western and Japanese pathologists (*N* = 21), 14 lesions were classified as adenocarcinoma by Western pathologists, compared to 19 of the same lesions by those from Japan [45]. The ‘Vienna classification’ was proposed for standardisation. Although this classification system is widely used, it is qualitative and does not take disease progression into account.

### Molecular and genetic advances

Mutations within Barrett’s oesophagus segments develop over time even in non-dysplastic epithelium. Increased clonal diversity is a marker for progression to dysplasia [46]. Clonal populations are stable over time, indicating that the dysplastic potential of Barrett’s oesophagus may be pre-determined. This implies that if this potential could be accurately determined at the index endoscopy, then further surveillance or therapies could be targeted only to those with dysplastic potential [47, 48].

Reflux injury appears to be key in the pathogenesis to OAC. Duodenogastric acid and contents reflux including unconjugated bile acids such as deoxycholate, can upregulate pro-inflammatory cytokines including IL-1β, IL-6, IL-8, and related transcription factors, notably NFκB that are relevant to metaplasia, dysplasia and cancer, and resistance to apoptosis [49–51].

Studies have demonstrated that there is a loss of both tumour suppressors *p16* and *p53* [52, 53]*.* As well as point mutations, aneuploidy has an important role in the progression of Barrett’s oesophagus to OAC [54–56]. Su et al*.* [57] reported that variants in the major histocompatibility complex (MHC) locus and at chromosome 16q24.1 locus, near *FOXF1,* predispose to Barrett’s oesophagus. Levine et al. [58] identified three gene associations (*FOXF1*, 19p13 in *CRTC1*, 3p14 near *FOXP1*) that were implicated in oesophageal cancer development from Barrett’s oesophagus.

Both Barrett’s oesophagus and OAC are characterised by a loss of heterozygosity (LOH), aneuploidy, specific genetic mutations, and clonal diversity [59]. There is currently ongoing research into establishing panel genetics and epigenetics involved in Barrett’s oesophagus and its progression to OAC.

### Field effect

Numerous previous studies from the 1980s and 1990s have demonstrated multifocal high-grade dysplasia and adenocarcinoma in Barrett’s oesophagus specimens, suggesting a field effect for carcinogenesis [60, 61]. The term ‘field of cancerisation’ was initially used by Slaughter et al. [62] when studying oral cancer. The authors summarised findings related to cancer developing in multifocal areas of precancerous change, and abnormal tissue that surrounds tumour cells. Mechanisms that lead to an impairment of DNA damage repair mechanisms in the tumour can show a field effect on the surrounding mucosa, which is facilitated by the adjacent inflammatory processes [63]. Reflux-related changes in the distal oesophagus increase the population of regulatory T cells and activate myeloid dendritic cells [64]. This cytokine increase supports epithelial mesenchymal transition in the distal oesophageal mucosa [65].

The loss of the *p53* tumour suppressor gene has been studied extensively in patients who have already developed adenocarcinoma [66–68]. More research is necessary in *p53* behaviour in patients who have Barrett’s oesophagus without adenocarcinoma in vivo subjects [69].

### Recognised biomarkers

There is a need for reliable biomarkers to aid diagnosis and distinguish which Barrett’s oesophagus patients are at risk of developing OAC [70]. This would potentially reduce the number of patients required to undertake endoscopy. Multiple ongoing studies into establishing biomarkers reflects the fact that Barrett’s oesophagus needs a clinically validated prognostic tool to aid in defining risk [11]. Technology such as gene expression analysis, epigenetics and proteomics have been utilised to establish biomarkers in Barrett’s oesophagus and OAC [71]. Trefoil Factor 3 (TFF3) has been used in the cytosponge device providing presence or absence of Barrett’s oesophagus with good affect (Table 3).

**Table 3:** Summary of molecular biomarkers [70, 74]

Biomarker	Sample size	Baseline histology	Endpoint
DNA abnormalities			
Aneuploidy/tetraploidy	322	SIM, indefinite, LGD	OAC
Biomarker panels			
Loss of heterozygosity	243	SIM	OAC
Expert LGD, *Aspergillus oryzae,* lectin protein	380	SIM, indefinite, LGD	OAC
8 gene panel methylation	195	SIM	HGD/OAC
Epigenetics			
*p16* methylation	53	SIM, LGD	HGD/OAC
Tumour suppressor loci			
*p53* staining	48	LGD	HGD/OAC
*p53* loss of heterozygosity	256	SIM, indefinite, LGD	OAC
Cell cycle markers			
Cyclin A	48	SIM	HGD/OAC
Cyclin D1	307	SIM	OAC
Clonal diversity			
Clonal diversity measures	239	SIM	OAC
Proliferation			
MCM2	27	SIM	OAC
Serum biomarkers			
Selenoprotein P	361	Variable	OAC
Leukocyte telomere length	300	Variable	OAC

*SIM* specialised intestinal metaplasia, *LGD* low-grade dysplasia, *HG*: high-grade dysplasia, *OAC* oesophageal adenocarcinoma, *MCM2* minichromosome maintenance protein 2

Mutations within *p16* which result in clonal expansion have been found to be one of the earliest changes in Barrett’s oesophagus [72, 73] *p16* unfortunately has been not been shown to be associated with the grade of dysplasia [73]. Wong et al*.* [73] have interrogated other biomarkers for Barrett’s oesophagus which include the cell cycle markers Cyclin A and D. When present, they indicate inactivation of *p105-Rb* which restricts the cell's ability to replicate DNA [73]. Studies are still preliminary.

Weaver et al*.* [47] assessed clonal structure using whole-genome sequencing across common mutations in Barrett’s oesophagus, HGD and OAC samples. The authors established the presence of *SMAD4* clearly demonstrated risk of progression to cancer. It was, however, found at a low frequency within OAC tissue (13%). In this study, *p53* was found to be mutated in both HGD (72%) and OAC (69%) samples, but only 1 case (2.5%) of ‘never-dysplastic’ oesophagus. This builds on previous work which has demonstrated a significant increase in progression to OAC in those samples containing defects within the *p53* gene [74].

The ideal biomarker has to be cost-effective, minimally invasive and superior to current diagnostics with multiple biopsies. Further research is necessary to build on risk stratifying Barrett’s oesophagus and aiding in prompt LGD and HGD diagnoses in these patients to reduce the burden of OAC developing in these patients. Identifying those at low risk would enable better risk stratification of patients, which could direct resources to patients who need treatment most. This would also eliminate unnecessary endoscopy. No clear data are available supporting the use of biomarkers or clinical features, which can sub-select those at higher risk of progression, other than an expert diagnosis of LGD.

### Screening

Screening is based on the presence of multiple risk factors including chronic GORD, male sex, white race, patients over 50 and a high BMI [17, 75]. A large number of asymptomatic patients may miss the opportunity for their cancers to be detected early. Lagergren et al. [76] found that up to 40% of patients with OAC had no history of chronic GORD. Furthermore, sampling and diagnostic errors with inter-variable pathological discrepancies results in a reduced effectiveness of screening [44].

The gold standard method of screening is visual OGD inspection and four quadrant biopsies of mucosal irregularities in salmon-coloured mucosa above the OGJ at every 1–2 cm interval using Seattle protocol [77]. This has been clarified by NICE guidelines on management of dyspepsia published in 2014 [78]. They recommend that OGD should be considered if a patient with GORD has risk factors including older age, male gender and a history of reflux or others such as a long duration of symptoms, increased frequency of symptoms, previous oesophagitis, previous hiatus hernia, oesophageal stricture or oesophageal ulcers.

Saad et al. [79] established that standard brush cytology demonstrated a high diagnostic accuracy for HGD/OAC (sensitivity 90%), moderate sensitivity for Barrett’s (60 vs. 92%) and low sensitivity for LGD (20 vs*.* 97%) compared with histology. Alexander et al. [80] commented that although brush cytology compliments histology there is an added increased cost with no true improvement. Standard OGD is expensive and associated with a small risk of complications such as bleeding, perforation, aspiration and cardiopulmonary events [81]. Since OGD is not a suitable method for screening of large populations there exists a need for alternative, cheap, widely available, and an accurate method of screening [82].

The ACG has recommended unsedated transnasal endoscopy as an alternative to traditional endoscopy for screening in Barrett’s oesophagus. Unsedated transnasal endoscopy is performed using an ultrathin endoscope using topical anaesthesia. Shariff et al. [83] reported that unsedated transnasal endoscopy was safer with fewer procedure- and sedation-related complications compared to standard OGD. Jobe et al. [84] established the sensitivity for detection of columnar-lined oesophagus was 98% and of intestinal metaplasia was 91% and specificity was 100% with unsedated transnasal endoscopy compared to standard OGD.

Non-invasive methods such as the cytosponge or capsular endoscopy have been utilised to screen for specialised intestinal metaplasia but do not alter the difficulty of sub-selecting a population with increased prevalence of specialised intestinal metaplasia or oesophageal adenocarcinoma. Cytosponge is a mesh surrounded by a gelatin capsule attached to a string which is passed transorally [85]. The capsule dissolves in the proximal stomach 5 min post-ingestion, expanding the mesh. The sample containing cytological specimen is stained with Trefoil Factor 3 (TFF3), which is a biomarker for specialised intestinal metaplasia. Kadri et al. [86] found that the cytosponge with TFF3 had a sensitivity of 73.3% (95% CI 44.9–92.2%) and specificity of 93.8% (95% CI 91.3–95.8%) for detecting Barrett’s ≥1 cm of circumferential length. Heberle et al. [87] carried out a cost-analysis and established that screening GORD patients with cytosponge and following up positive results with OGD for confirmation reduced cost by 27–29% when compared with screening by OGD alone.

Capsule endoscopy allows oesophageal visualisation using wireless cameras without obtaining a biopsy. Capsule endoscopy has reported a sensitivity and specificity of 77 and 86%, respectively, compared to standard OGD, but just 73% specificity compared with histologically confirmed specialised intestinal metaplasia in a meta-analysis of 9 studies (*n* = 618) [88].

Liquid biopsies utilising blood samples and extracting circulating microRNAs expressed in disease are gaining promise as a screening tool. Bus et al. [89] profiled circulating microRNAs in patients with Barrett’s. The authors found that in 41 patients with Barrett’s and 15 controls, a panel of 4 circulating miRNAs (miRNA-95-3p, -136-5p, -194-5p, and -451a) distinguished Barrett’s oesophagus from controls with a sensitivity and specificity of 78 and 86%, respectively.

### Surveillance

The primary aim of surveillance of Barrett’s oesophagus is to identify dysplasia and malignancy before distant disease has advanced. OAC usually presents with advanced disease as a result of early lymphovascular submucosal invasion [90]. OGD remains the primary method of surveillance using the Seattle protocol [91]. The frequency of surveillance is determined by the degree of dysplasia encountered at biopsy which are subsequently then classified as per the Vienna classification [45]. It is important to note that surveillance endoscopy should be performed in patients whose reflux symptoms are controlled, reducing the probability of reactive changes interfering with pathological interpretation [92].

In the United Kingdom, for non-dysplastic disease (metaplasia only), surveillance every 2–5 years is offered to patients. Worldwide guidelines differ with regards to differing Barrett’s oesophagus segment length. The BSG guidelines state that endoscopy should be repeated 3–5 years if the maximal length is less than 3 cm, and every 2–3 years if above or equal to 3 cm [16]. Evidence for improved outcomes from surveillance is weak and remains the subject of debate. The UK multicentre BOSS trial aims to compare the benefits of 2-yearly surveillance endoscopy against endoscopy on an ‘at need’ basis only.

Some literature does demonstrate a survival advantage in patients with Barrett’s undergoing surveillance. El Serag et al. [93] found that patients diagnosed with OAC during surveillance were detected at an earlier stage (stage 0–1: 74.7 vs. 56.2; *p* < 0.001), survived longer (median 3.2 vs. 2.3 years; *p* < 0.001), and had lower cancer-related mortality (34.0 vs. 54.0%, *p* < 0.0001) compared with those not in surveillance. As the natural course of Barrett’s in unknown, and surveillance is expensive and time-consuming, surveillance has been the subject of much enquiry [94].

A cost-effective analysis of surveillance in patients with non-dysplastic disease at 5 yearly intervals was found not to be cost effective and that unless the annual progression rate to adenocarcinoma were 1.9% then a QALY (Quality Adjusted Life Year) threshold of <$50,000 could not be achieved [95]. This finding is similar to previous studies highlighting that surveillance in this group is not cost-effective [91]. Findings such as inflammation and ulceration considered to be indefinite for dysplasia may evolve as a result of erosive oesophagitis. Acid suppression should be offered and a repeat endoscopy should be offered in 6 months [16].

If a histological finding of LGD is encountered, a repeat endoscopy should be performed at 6 months. If this confirms the diagnosis, discussions should be had regarding endoscopic surveillance (every 6 months for 2 years, annually thereafter) vs. eradication therapy (radiofrequency ablation, endoscopic segmental resection, photodynamic therapy, spray cryotherapy). The absolute benefit of eradication therapy for LGD is not certain, since the progression rate of LGD to OAC is low (approx. 5 per 1000 patient years) [96].

**Fig. 7:**
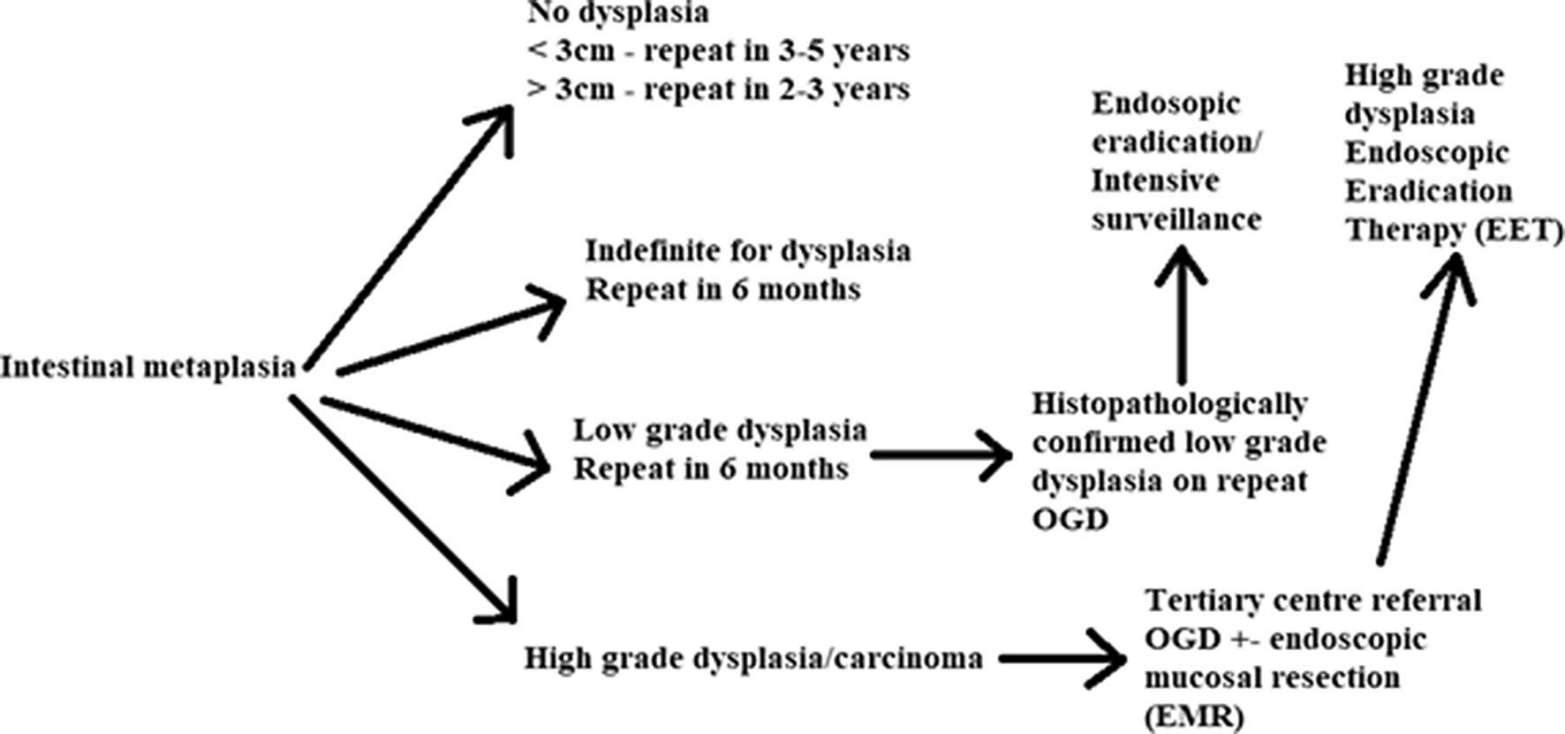
Surveillance and management for dysplasia (LGD/HGD)

When HGD is encountered, two expert GI pathologists should analyse the samples and patients should be referred to a tertiary centre for consideration of repeat endoscopy, biopsies, endoscopic mucosal resection and eradication therapy (Fig. 7) [16].

### Management

Patients with non-dysplastic Barrett’s oesophagus should be on acid suppression therapy. However, there is no convincing evidence that this reverses specialised intestinal metaplasia [9]. LGD should be managed with endoscopic surveillance biopsies with endoscopic resection [17]. Endoscopic radiofrequency ablation (RFA) has been utilised in LGD patients. An American multicentre retrospective cohort study discovered a 0.8% progression rate amongst patients with LGD diagnosed by expert pathologists and treated with RFA compared with 6.6% in the control surveillance group [97]. No clear data are available supporting the use of biomarkers or clinical features which can sub-select those at higher risk of progression, other than an expert diagnosis of LGD. Patients with multifocal areas of dysplasia may have an increased risk of progression, as many patients with persistent LGD over time [98].

Upfront ablation has been shown to be a cost-effective strategy compared to intensive surveillance. A cost-effectiveness analysis model stated that ablation with RFA in patients with LGD is more cost-effective than surveillance if ablation permanently eradicated LGD in more than 28% of patients, without the need for further surveillance in this group [99]. Previous BSG and AGA guidelines did not recommend ablation for LGD. The SURF (Surveillance vs. Radio Frequency ablation) trial randomised patients with LGD to RFA (*n* = 68) vs. intensive surveillance (*n* = 68). Results published in 2014 found that ablation reduced the risk of progression to HGD or adenocarcinoma from 26.5% in the control arm to 1.5% in the RFA arm (95% CI 14.1–35.9%, *p* > 0.001); 88.2% of intestinal metaplasia was eradicated and 92.6% of dysplasia using RFA, vs. rates of 0 and 27.9% in the control arm [100].

Patients with HGD should be offered options including endoscopic therapies including RFA ± endoscopic mucosal resection, surgical resection, or intensive surveillance. There should be discussions involving the patient’s fitness for surgery and the patient’s desires. Ablative therapies treat entire Barrett’s oesophagus segments and surgery should only be necessary for patients with risk factors for lymph node metastases. Current standards reserve oesophagectomy for patients with T1b invasion (submucosal invasion), multifocal carcinoma or lesions that are not amenable to endoscopic resection [101].

A recent cost-effectiveness analysis was conducted for endoscopic eradication therapy (EET) for treatment of all grades of dysplasia in Barrett’s oesophagus patients [102]. EET for patients with LGD and HGD arising in Barrett’s oesophagus was deemed as cost-effective compared to endoscopic surveillance alone (lifetime £3,006 per QALY gained) by Pollit et al. [102] The authors further concluded that as the time elapses, the treatment becomes more cost-effective. The 5-year financial impact to the UK National Health Service (NHS) of introducing EET is £7.1 m [102, 103].

Photodynamic treatment (PDT) using 5-aminolevulinic acid and porfimer sodium has been shown to be inferior to RFA. In a multicentre study by Overholt et al., 13% of patients progressed to adenocarcinoma despite treatment [104]. No studies with long-term follow-up have shown an improved overall survival of PDT vs. oesophagectomy [105]. The current guidelines state that patients should continue ongoing surveillance. There is no long-term data (>5 years) on the recurrence of intestinal metaplasia or dysplastic changes in squamous epithelium.

## Imaging modalities in Barrett’s oesophagus

The need for an alternative non-invasive method of screening and/or surveillance could be highly beneficial reducing waiting times, alleviating patient fears and reducing future costs in modern healthcare. Thorough endoscopic assessment and biopsies are key to a diagnosis of Barrett’s oesophagus and subsequent surveillance. Most endoscopists appreciate quadrantic biopsies are time-consuming and advanced imaging would be greatly beneficial.

### High-resolution endoscopy

High-resolution light endoscopy enhances mucosal visualisation combining pixelated endoscopes (up to 1,000,000) with high definition screens. Studies have demonstrated a greater sensitivity in the detection of early neoplastic lesions when compared to standard endoscopy [106].

### Chromoendoscopy

Chromoendoscopy is a diagnostic tool where a chemical substance is sprayed onto the mucosal surface to highlight specific areas of epithelia. The stains used can be subdivided into ‘absorptive’ (acetic acid, methylene blue, lugol solution) and ‘non-absorptive’ (indigo carmine) contrast stains. Advanced imaging modalities magnify the view and this subsequently increases the probability of finding suspicious lesions. Studies have demonstrated an increased diagnostic yield using Chromoendoscopy in recognising dysplasia in Barrett’s compared to random biopsies [107].

Acetic acid spraying with targeted biopsies has shown an increased detection rate of dysplasia and OAC, even with white light standard endoscopy [108]. Longcroft-Wheaton et al*.* [109] demonstrated that acetic acid chromoendoscopy yielded a 95.5% sensitivity and 80% specificity for the detection of OAC. Indigo carmine used as a non-absorptive contrast stain has shown to be highly sensitive (83%) and specific (88%) for HGD [110]. The agent is currently not able to differentiate between specialised intestinal metaplasia and dysplasia [111].

### Autofluorescence

Autofluorescence imaging endoscopy utilises short wavelengths of light to stimulate endogenous substances (nicotinamide adenine dinucleotide (NADH), collagen, aromatic amino acids and porphyrines) in tissue to emit fluorescent light of a longer wavelength [112]. This interrogates the tissue at depth and aids interpretation of vasculature and topography [113]. Studies have not demonstrated the superiority of this method compared with high resolution endoscopy alone for the detection of dysplasia [114, 115]. Consequently, autofluorescence guided biopsies are not currently employed in hospitals in the United Kingdom.

### Narrow-band imaging (NBI)

Narrow-band imaging allows visualisation of the superficial mucosa and vasculature without the need for any additional dyes. The system illuminates the mucosa with blue and green wavelength light, thus demonstrating tissue vasculature. A meta-analysis by Mannath et al. [116] evaluated 446 patients. For diagnosing HGD, the pooled sensitivity and specificity were 0.96 (95% confidence interval [CI] 0.93–0.99) and 0.94 (95% CI 0.84–1.0) on a per-lesion analysis with similar results on per-patient analysis. For specialised intestinal metaplasia characterisation, the pooled sensitivity and specificity were 0.95 (95% CI 0.87–1.0) and 0.65 (95% CI 0.52–0.78) on a per-lesion analysis. The authors concluded that magnified NBI is accurate with high diagnostic precision for diagnosis of HGD in Barrett’s oesophagus. They further commented that NBI has high sensitivity but poor specificity for characterising specialised intestinal metaplasia.

### Optical coherence tomography (OCT)

Optical coherence tomography (OCT) utilises electromagnetic (EM) waves to generate images based on the detection of reflected light. Resolutions up to 10–25 µm enables the identification of microscopic features such as lymphovascular structures [117]. Robles et al. [118] interrogated 19 studies (17 in vivo; 2 ex vivo). The authors found an excellent diagnostic yield for specialised intestinal metaplasia detection but not for dysplasia. Evans et al*.* [119] only demonstrated an 83% sensitivity and 75% specificity between differentiating HGD and OAC.

### Confocal fluorescence microendoscopy

This diagnostic tool images fluorophores within the cell microstructure and generates a histological image [120]. Kara et al. [121] evaluated 63 patients in ex vivo samples. The authors concluded that a differentiation could be made between Barrett’s oesophagus and HGD, but that a diagnosis of dysplasia needed histological guidance.

Curvers et al. [122] concluded that the above enhanced imaging techniques may be no better than using high-quality white light imaging. Furthermore, these methods fail to achieve the aim of replacing random biopsies and histology for diagnosis. This opens the door for innovative diagnostic, screening and surveillance modalities to be explored in the field of Barrett’s oesophagus and its subsequent transformation to OAC.

## Future developments

High-quality white light imaging with OGD and biopsy remains the gold standard of diagnosis and surveillance. Dysplasia and *p53* accumulation appear to be an earlier and more sensitive markers of malignant potential in Barrett’s oesophagus. There remains substantial inter-observer variability with regards to the grading of dysplasia between pathologists. Improving the diagnosis of dysplasia and categorising patients at an early stage of disease is highly encouraged. There is an additional need for reliable biomarkers in being able to aid diagnosis and potentially reduce the number of patients required to undertake endoscopy [70]. Multiple ongoing studies into establishing biomarkers reflects the fact that Barrett’s oesophagus needs a clinically validated prognostic tool to aid in defining risk [123]. If a marker were to be isolated that indicated the propensity to dysplasia, this could establish why some patients progress to dysplasia and, therefore, aid in identifying preventative measures.

Very few potential diagnostic and prognostic biomarkers have been shown to be reproducible and robust [70]. Immunohistochemistry studies of nuclear *p53* expression in patients with Barrett’s oesophagus have shown to improve inter-observer variability in diagnosing dysplasia and can predict progression risk with an OR of 3–8 [124, 125]. Many studies have investigated genetics and epigenetics in relation to Barrett’s oesophagus and its progression to OAC [126–128]. However, a paucity of evidence for other epigenetic markers and the absence of robust validation methods limit the conclusions that can be drawn from such literature [129].

### Vibrational spectroscopy

Vibrational spectroscopy techniques such as Fourier-transform infrared (FTIR) spectroscopy or Raman spectroscopy (RS) are used to study interactions of light with biological materials and are relatively novel [130]. Advantages of spectrochemical analysis are its low cost, minimal sample preparation, non-destructive nature and substantially accurate results [131]. Biospectroscopy for disease diagnosis and screening is possible in a wide range of conditions including cancer. Further substantial prospective trials are necessary to delineate whether biospectroscopy has the ability to identify the small number of at-risk individuals amongst the large number not requiring follow-up [132].

Vibrational spectroscopic techniques have been used to delineate classification of oesophageal tissue from Barrett’s oesophagus through to OAC. Old et al*.* [133] identified key biochemical differences categorised spectral signatures using ATR-FTIR spectroscopy in ex vivo samples: high glycogen content was seen in normal squamous tissue, high glycoprotein content was observed in glandular Barrett’s oesophagus tissue, and high DNA content was observed in dysplastic tissue/OAC samples. Classification of normal squamous samples vs. 'abnormal' samples (any stage of Barrett's oesophagus) was performed with 100% sensitivity and specificity. Neoplastic Barrett's (dysplasia or OAC) oesophagus was identified with 95.6% sensitivity and 86.4% specificity [133].

In vivo RS has been utilised in the field of OAC. Sensitivities and specificities of up to 95% have been established for an in vivo diagnosis of HGD and OAC using multimodal image-guided Raman endoscopy techniques [134, 135]. Non-endoscopic approaches remain to be validated in large populations in terms of accuracy and cost effectiveness. Novel non-invasive markers identifiable in biofluids would be ideal for widespread application, but are currently not available. The use of vibrational spectroscopy in biofluids has been shown to be promising in categorising disease processes from Barrett’s oesophagus to OAC [136, 137]. This needs further, prospective multicentre studies for clinical validation.

## Conclusion

Barrett’s oesophagus is the only known precursor to OAC with a population prevalence of around 1–2% [3]. Established risk factors include older age, male gender and a history of reflux symptoms [8]. Although guidelines on the screening and surveillance exist in Barrett’s oesophagus, the current strategies are inadequate as more than 90% of patients diagnosed with OAC do not have a preceding diagnosis of Barrett’s oesophagus [138]. Furthermore, the annual risk for developing OAC has been shown in large population studies to be as low as 0.16% [139].

OGD is the gold standard method in screening for Barrett’s oesophagus. This invasive method is expensive with risks associated negating its use as a current screening tool. The need for an alternative, non-invasive method of screening and/or surveillance would be highly beneficial in reducing waiting times, alleviating patient fears and reducing future costs in worldwide healthcare sectors. Vibrational spectroscopy in biofluids has shown promise in categorising disease processes from Barrett’s oesophagus to OAC [136, 137]. This needs further, prospective multicentre studies for clinical validation.
